# The economic impact of stroke in The Netherlands: the €-restore4stroke study

**DOI:** 10.1186/1471-2458-12-122

**Published:** 2012-02-13

**Authors:** Mitchel van Eeden, Caroline M van Heugten, Silvia MAA Evers

**Affiliations:** 1 CAPHRI, Research School for Public Health and Primary Care, Department of Health Services Research, Faculty of Health, Medicine and Life Sciences, Maastricht University, P.O. Box 616, 6200, MD Maastricht, The Netherlands; 2 MHeNS, School for Mental Health & Neuroscience, Maastricht University, Maastricht, The Netherlands; 3 Department of Psychiatry & Neuropsychology, Faculty of Health, Medicine & Life Sciences and Department of Neuropsychology & Psychopharmacology, Faculty of Psychology & Neuroscience, Maastricht University, Maastricht, The Netherlands

## Abstract

**Background:**

Stroke has a considerable socio-economic impact worldwide and is the leading cause of disabilities in the Western world. Economic studies of stroke focus merely on physical aspects and clinical interventions. To our current knowledge there is no comprehensive economic study investigating the economic impact of stroke including psychological and social aspects. The €-Restore4Stroke project, part of a large comprehensive research programme Restore4Stroke, aims to investigate the total economic impact of stroke in the Netherlands.

**Methods:**

Two trial-based economic evaluation studies will be conducted within the €-Restore4Stroke project: one focussing on a self-management intervention and one on an augmented cognitive behavioural therapy intervention. Both include cost-effectiveness analyses and cost-utility analyses as primary research methods. Furthermore, a cost-of-illness study investigating costs after stroke attached to a cohort study and a record linkage study in which four databases are linked to investigate patterns of health care consumption before and after stroke, are embedded in €-Restore4Stroke. All studies will be performed from a societal perspective. The primary outcome measure for the cost-effectiveness analysis is the increase in health status on the primary outcome scales. Within the cost-utility analysis, the primary outcome measure is quality-adjusted life years (QALYs) for which an indirect preference-based technique will be used. In the self-management study we will also look at the estimation of health effects on informal caregivers. Cost outcomes in the cost-of-illness study will be computed with a cost questionnaire and linkage of several databases will be used to derive outcomes in the record linkage study,

**Discussion:**

€-Restore4Stroke will provide new insights and evidence for the economic impact of psychosocial consequences after stroke. Besides being innovative in various ways (i.e. focussing on the chronic phase after stroke and including personal factors as possible determinants of long-term re-integration including quality of life in a prospective longitudinal design), a major strength of €-Restore4Stroke is that we include impact on informal caregivers. The outcomes of this study will provide health care decision makers with valuable and necessary information regarding stroke care related decisions.

**Trial registration:**

NTR3051 (RCT Self-management), NTR2999 (RCT Augmented Cognitive Behavioural Therapy)

## Background

Stroke has a major socio-economic impact worldwide and is the leading cause of disabilities in the Western world. It is estimated that the worldwide prevalence of stroke is 0.2% of the world population. Of the people who suffer from a stroke, 30% die, 30% are left functionally disabled and 40% have a successful recovery with minor to no disabilities [1]. It is estimated that by 2023 the absolute number of patients experiencing a first stroke will increase, in comparison with 1983, by 30% worldwide [2]. The disease impact of stroke is considerable on stroke patients and on their informal caregivers. More than 50% of the stroke survivors return to their homes after being discharged from the hospital [3]. At home, these survivors and their informal caregivers have to deal with the long term consequences of stroke, which are seen in physical, psychological and social areas of functioning [4,5].

**Figure 1:**
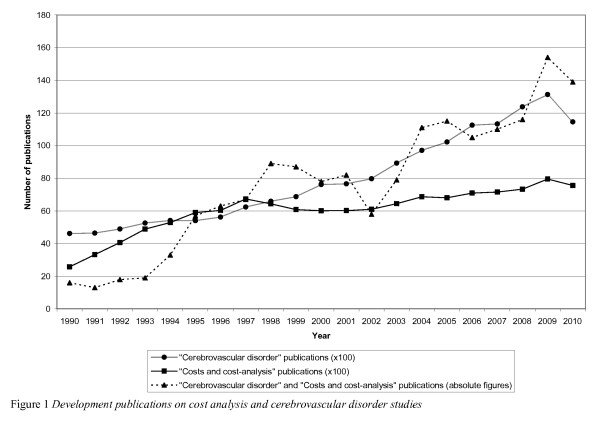
**Development publications on cost analysis and cerebrovascular disorder studies**.

Not only the clinical, but also the financial burden of stroke is considerable [6]. Currently, approximately 3-4% of total health care expenditures in Western countries are spent on stroke [7]. On the one hand, governments are cutting down on expenditures for health care, while on the other hand the health care sector is facing an increased demand for stroke care, due to demographic changes (e.g. an aging population) and changing attitudes towards health care (e.g. more demanding society; value for money) [8]. The growing demand for stroke care and the limited resources available for health care, have led to an increased interest in the economic aspects of stroke. The last few years have shown a remarkable increase in the publication of economic studies on stroke [7]. A Pubmed search combining "Cerebrovascular disorders" and "Costs and cost-analysis" shows that since 1990 the number of stroke studies including costs have increased in both absolute and relative terms. Figure 1 shows this development from 1990 until 2010, the last year for which complete data were available.

In general, these economic studies of stroke focus primarily on physical aspects and clinical care [9-14]. It is known that recovery from stroke also depends on psychological and social aspects. To our current knowledge there is no comprehensive economic study that investigates the economic impact of stroke including psychological and social aspects. This article presents the design and methods of a study which aims on investigating and determining the economics of psychosocial care after stroke. The present study, €-Restore4Stroke (pronounce as e-Restore), is part of a Dutch national consortium called Restore4Stroke. Within Restore4Stroke, four large studies are executed aiming to improve the quality of life of stroke patients and the concurrent effects on their informal caregivers. Three of these studies focus more on outcomes while the fourth, €-Restore4Stroke, is designed, among other things, to investigate the economic aspects of the other three studies.

### Restore4Stroke

In addition to €-Restore4Stroke, the following three studies are embedded in Restore4Stroke: two randomized controlled trials (RCTs) and a cohort study. The first RCT, Restore4Stroke Self-Management (referred to as Self-Management study), aims at enhancing self-management in stroke patients and their informal caregivers. The second RCT, Restore4Stroke Augmented Cognitive Behavioural Therapy (referred to as the Augmented CBT study), aims at decreasing depression and anxiety complaints in stroke patients. With the third study, the Restore4Stroke Cohort study (referred to as the Cohort study), the course of quality of life in stroke patients and their informal caregivers is investigated, and factors predicting quality of life, including pre-stroke health situation factors, stroke-related factors, personal factors and partner factors are determined. In this design article we will focus on the economic aspects of all four studies, for further details on the other aspects of the two RCTs and the cohort we refer to publication elsewhere.

For current status updates you can also visit http://www.restore4stroke.nl.

All studies described in this article are approved by a legally recognized medical ethics committee. The Self-Management study was approved by the Medical Ethics Committee Utrecht Medical Center. The Augmented CBT study was approved by the Committee On Research involving Human Subjects (CMO) Arnhem/Nijmegen. The Cohort study was approved by the Committee Research involving Human Subjects (CMO) St. Antoniusziekenhuis, Nieuwegein. All ethics approval committees are located in The Netherlands.

### €-Restore4Stroke

**Figure 2:**
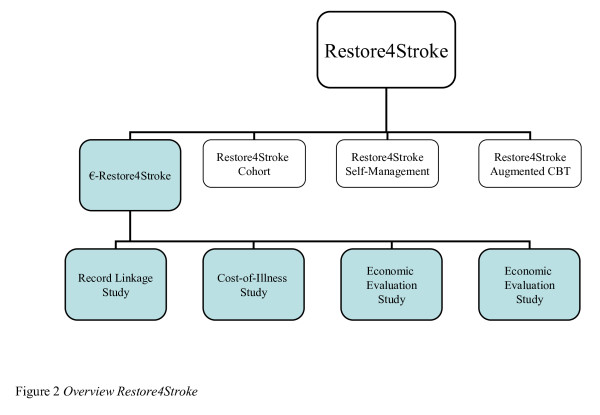
**Overview Restore4Stroke**.

The €-Restore4Stroke project consists of four sub-studies. Attached to both RCTs, an economic evaluation study will be performed. Economic evaluation studies compare two or more health care interventions and investigate the relationship between costs and effects. Furthermore, a cost-of-illness study will be performed for the Restore4Stroke cohort. Cost-of-Illness studies aim at determining the total costs of a disease for society. Finally, the fourth sub-study within €-Restore4Stroke is a record linkage study. In this study, we will focus on changes in health care consumption 5 years before and 5 years after stroke through register and database linkage. An overview of all four studies is provided in Figure 2.

## Methods

### Aim and research questions

The overall €-Restore4Stroke aim is to fully capture the economic impact of psychosocial care after stroke. Looking at the 4 subprojects embedded in this study, the following research questions will be answered:

• From a societal perspective, what are the costs and effects of the Self-Management intervention in comparison with an education intervention?

• From a societal perspective, what are the costs and effects of the Augmented CBT intervention in comparison with a computerized cognitive training intervention?

• How do patterns of care received after stroke (in terms of health care costs, productivity costs, costs of informal care) change during the time of the follow-up period of the Cohort study and what is their impact on the patient (in terms of health-related quality of life, life satisfaction and emotional functioning) in economic terms as well?

• How do patterns of health care consumption after stroke (in terms of health care costs, productivity costs, costs of informal care) change during the 5 years after stroke, in comparison with the 5 years before stroke and what is the economic impact of these changes from a societal perspective (record linkage study)?

### €-Restore4Stroke outcome measures

All studies included in the €-Restore4Stroke project will be performed from a societal perspective meaning that all costs and outcomes of both the interventions and comparators will be included. Within the Restore4Stroke programme, Quality of life (QOL) is considered both from a general Health-Related Quality Of Life (HRQOL) and domain-specific QOL perspective. The general HRQOL perspective is operationalised as disease-specific HRQOL measured with the Stroke Specific Quality of Life (SS-QoL [15]), and generic HRQOL measured with the 5-dimensional EuroQol (EQ-5D [16]). The domain-specific perspective consists of the domains: participation measured with the Utrecht Scale for Evaluation and Revalidation Participation (*Utrechtse Schaal voor Evaluatie en revalidatie - Participatie *or USER-p [17]), emotional functioning measured with the Hospital Anxiety and Depression Scale (HADS [18]) and subjective well-being measured with 3 satisfaction questions. An overview of the primary and secondary outcome measures of all studies embedded in the Restore4Stroke programme is presented in Table 1.

**Table 1:** Outcome measures Restore4Stroke (x: cost questionnaires, p: primary outcome measure, s: secondary outcome measure)

	Self-Management study				Augmented CBT study				Cohort study				
	T0	T1	T2	T3	T0	T1	T2	T3	T1	T2	T3	T4	T5

***Primary outcomes €-Restore4Stroke***													

Cost Questionnaire	x	x	x	x	x	x	x	x		x^a^	x^a^	x^a^	x^a^

CarerQol Questionnaire	s	s	s	s									

***Primary outcomes Restore4Stroke***													

EQ-5D^b^	s	s	s	s	s	s	s	s		s	s	s	s

HADS^c^	s	s		s	p	p	p	p	s	s	s	s	s

USER-P^d^	s	s		s	s	s	s	s	p	p	p	p	p

SS-Qol-12^e^	s	s		s	s	s	s	s		s	s	s	s

Life satisfaction	s	s		s	s	s	s	s		s	s	s	s

***Other outcomes***													

UPCC^f^	p	p	p	p									

VAS^g^	s	s		s									

ECSI^h^	s	s		s									

PSDRS^i^					s	s	s	s					

GSES^j^	p	p	p	p									

GAS^k^					s	s	s	s					

^a^The cost questionnaire used in the cohort study is a short questionnaire (11 items) compared to the cost questionnaire used in the Self-Management and augmented CBT Study (19 items)

^b^5 Dimensional EuroQol

^c^Hospital Anxiety and Depression Scale

^d^Utrecht Scale for Evaluation and Regabilitation Participation (*Utrechtse Schaal voor Evaluatie en Revalidatie - Participatie*)

^e^Stroke Specific Quality of Life

^f^Utrecht Proactive Coping Competence list

^g^Visual Analogue Scale

^h^Expanded Caregiver Strain Index

^i^Post Stroke Depression Rating Scale

^j^General Self-Efficacy Scale

^k^Goal Attainment Scale

### Designs

The study design is distinctive for each study and will be explained in the following section.

#### Self-Management study and Augmented CBT study

Both trial-based economic evaluations will involve a combination of a cost-effectiveness analysis (CEA) and a cost-utility analysis (CUA). Costs will be calculated in various ways. Effects will be presented as clinical outcomes, which are the primary outcome parameters of both the Self-Management study (i.e. the UPCC) and the Augmented CBT study (i.e. the HADS). In a CUA, costs are calculated in a similar way as in a CEA, but effects are usually expressed in quality-adjusted life years (QALYs) [19]. A QALY combines two distinct variables: quality or utility (the value which stroke patients attach to their current health status) and quantity (life years gained) of health. Utilities in both studies will be derived from the Five-Dimensional Euroqol (EQ-5D). The EQ-5D is chosen because it is a widely used quality of life instrument, also in the field of stroke. To estimate the incremental cost-effectiveness, the incremental cost-effectiveness ratio (ICER) will be calculated for both the CEA and CUA. If there are two alternative interventions, their difference in cost (incremental cost) is compared with their difference in outcomes (incremental effect) by dividing the former by the latter. This ratio is known as the ICER and is expressed as the incremental cost per point improvement on the primary outcome measure or otherwise costs per QALY.

In addition, in the Self-Management study we will include a questionnaire to investigate the quality of life of informal caregivers. Performing an economic evaluation from a societal perspective means including all relevant costs and effects, but economic evaluations of health care interventions usually treat patients as isolated individuals in determining the relevant health effects. Consequently, the quality of life of informal care is usually neglected in these studies. To address this issue, the CarerQOL [20] questionnaire will be included in the Self-Management study.

### Sample size calculation

The sample size calculation of the Self-Management study is based on a method presented by Jones et al. [21]. Based on two earlier intervention studies with the UPCC [22,23], a standardised difference was calculated representing the difference between the means/population standard deviation. The studies resulted in two different outcomes: a difference in means of .2 and .3 respectively between the groups on the UPCC and a mean SD of .35 and 0.6 respectively. Based on these studies, a standardised difference of .6 on the UPCC was used for the power calculation (.2/.35 = .57). Based on the method of Jones et al, 45 patients per group are needed based on an alpha of .5 and a power of 80%. Assuming a drop-out rate of 15%, 106 patients (2 × 53) will be recruited for this study.

Based on previous research, it has not been possible to conduct a power calculation on the HADS as the psychological intervention studies in the Cochrane review [24] all used different outcome measures and not the HADS. Hence, the sample size calculation for the Augmented CBT study will be based on other measures and is therefore identical to the sample size calculation for the Self-Management study (2 × 53 = 106).

### Setting and participants

Participants will be recruited from participating hospitals and rehabilitation centres in the Netherlands on the basis of case finding. For the Self-Management study, a minimum of 106 home-living mild stroke patients with re-integration problems and their informal caregivers on the basis of the USER-p will be recruited, through their own physicians or nurse practitioners. For the Augmented CBT study, a minimum of 106 patients who have depression and anxiety symptoms based on a cut-off score > 7 on the depression subscale of the HADS will be included. Patients will be recruited through a rehabilitation specialist, nurse practitioner or psychologist. For both studies, written informed consent for participation is obtained from both participants and their partners (if included) after recruitment.

### Timeline

**Figure 3:**
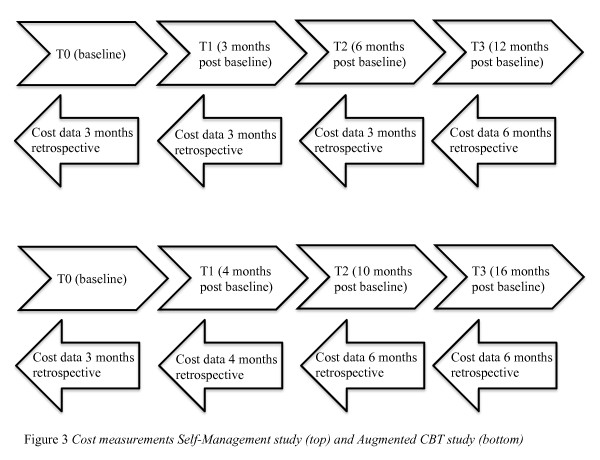
**Cost measurements Self-Management study (top) and Augmented CBT study (down)**.

For the Self-Management study, eligible patients and their informal caregivers will be randomized towards the Self-Management Intervention (SMI) or education intervention after baseline measurement T0. Both SMI and education intervention have a duration of 10 weeks and post-treatment measurement (T1) will take place at 3 months after T0. Two follow-up measurements (T2 and T3) will take place at 6 and 12 months after T0. For the Augmented CBT study, eligible patients will be randomized towards the Augmented CBT intervention the computerized cognitive training intervention after baseline measurement T0. Post-treatment measurements (T1) will take place 4 months from T0, and two follow-up measurements (T2 and T3) will take place 10 and 16 months from T0. Cost measurements for both studies are presented in Figure 3. At all measurement points, cost data will be asked retrospective varying from 2 to 6 months retrospective.

### Interventions

The SMI is designed as an effective proactive coping group intervention for both stroke patients and their informal caregivers, with a duration of ten weeks [25]. The intervention, including seven group sessions, will be provided by two specially trained therapists experienced in group counselling and working with brain injury patients. The sessions will be organised in the participating institutions. Group sessions are aimed at setting goals and attaining them. These goals relate to different themes, i.e. social support and relations, participation in society, and negative emotions. Six group sessions (two hours each) will be held in participating hospitals during the first six weeks and one booster session (for exchanging experiences and repeating theoretical concepts) will be held at 10 weeks from baseline. The control group will receive an education intervention aimed at providing passive information. One trained health care professional will provide three group sessions (one hour each) in the first 6 weeks concerning different themes (i.e. the brain, a stroke, and prevention of a recurrent stroke) and a booster session at week nine from baseline; all are held in the participating institutions.

The Augmented CBT treatment will focus on registering, recognising and altering mood, negative thoughts, cognitions and emotional symptoms that comprise depressive problems as well as anxiety. The intervention will be provided by an experienced health care psychologist in the participating institutions (10-12 sessions), where occupational therapists and/or movement therapists will be enrolled in the intervention as co-therapists (three to four sessions). The intervention is an individual therapy, in which patients are expected to participate in a total of 13 to 16 individualised sessions, in a time-span of four months. Test assistants or assistant psychologists working in the participating institution will assist execution of the control intervention programme, the computerized cognitive training programme CogniPlus, aimed at improving general cognitive functioning. Patients will participate in a total of 13 to 16 sessions in a time-span of for months, equal to the Augmented CBT intervention. The programme is self-supporting as most tasks can be executed without assistance.

### Outcome measures

In Figure 2 the outcome measures of the Restore4Stroke program in general and the four studies embedded in this program are shown. The primary outcome measure for the CEA will be the increase in health status as measured by the UPCC (Self-Management study) and the HADS (Augmented CBT study). Within the CUA, the primary outcome measures are QALYs for which an indirect preference-based technique will be used. In this technique, the patient's health status will be measured by means of the EQ-5D and weights that incorporate preferences from a general population sample will be used to calculate utilities. The EQ-5D is chosen because it is a widely used quality of life instrument (nationally and internationally). The EQ-5D contains five dimensions of health-related quality of life; namely mobility, self-care, daily activities, pain-discomfort and depression/anxiety [17]. Each dimension can be rated at three levels: no problems, some problems and major problems. The five dimensions can be summed into a health state. Utility values can be calculated for these health states, using preferences elicited from a general population, the so-called Dolan algorithm [26]. The utility values derived from the Dolan algorithm will be used to compute QALYs. The Dolan algorithm has been established using a general population from the UK. In 2006, a Dutch algorithm became available [27] using Dutch tariffs instead of UK tariffs to compute QALYs.

The utilities at the three time points will be used to compute a QALY score by means of the area under the curve method. Furthermore, the EQ-5D consists of a visual analogue scale (VAS) ranging from zero (worst imaginable health state) to 100 (best imaginable health state). The reliability and validity of the EQ-5D has been studied and established [28,29]. The base case analysis will use the individual utility score of the patient based on the EQ-5D.

To estimate the health effects on informal caregivers, the CarerQOL questionnaire has been chosen. This instrument was tested positively in terms of validity and feasibility in extensive research by Hoefman et al. [20]. The CarerQol questionnaire consists of two components, namely the CarerQol-7D and the CarerQol-VAS. The CarerQol-7D refers to seven dimensions, each represented by one question on the questionnaire. The seven dimensions are: fulfilment, relational dimension, mental health dimension, social dimension, financial dimension, perceived support and physical dimension The purpose of the CarerQol 7D is to help caregivers indicate their situation with respect to a particular dimension on one of three levels (*I have no/some/a lot of..*.). The CarerQol-VAS is a visual analogue scale, where caregivers can indicate how happy they feel, ranging from 0 (completely unhappy) to 10 (completely happy). The CarerQol questionnaire will be computed at every measurement point of the Self-Management study, T0-T3.

### Cost analysis

The following cost categories will be distinguished and included in this study: intervention costs, costs for the health care sector, patient and family costs and costs outside the health care sector. Intervention costs will include all the costs that contribute to the development and administration of the SMI and Augmented CBT. Health care sector costs are related, for instance, to general practitioner (GP) visits, hospital visits and medication. Patient and family costs concern, for instance, travel costs, costs of informal care, productivity losses and home adjustments. Costs outside the health care sector will be measured as productivity costs.

To measure the actual use of resources, data will be obtained using combined sources (registrations by professionals and a cost questionnaire for the patients conducted at al measurement points, T0 - T3, of both the Self-Management study and the Augmented CBT study). Resources used relating to the interventions will be based on the registered time all professionals spent on the treatment. All use of resources by the patient and their family, within and outside the health care sector will be measured by means of a cost questionnaire which will continuously record volumes of utilization during the follow-up period. The cost questionnaire has been designed especially for the participants in both RCTs, based on existing questionnaires [30]. The questionnaire consists of 20 items and will cover three areas of expenses, namely expenses of (inability to perform) daily activities, expenses of health care consumption and expenses of help received (material and immaterial).

### Valuation

The valuation of health care costs, patient and family costs will be based on the updated Dutch manual for cost analysis in health care research [31]. This manual recommends using standardised cost prices. In brief, the manual recommends that prices of informal care should be based on shadow prices for unpaid work (meaning a standard cost price based on general hourly wages). Costs of transport will be calculated as the mean distance per destination multiplied by costs per kilometre. Costs of medication will be calculated using prices based on the Daily Defined Dosage (DDD) taken from the Dutch Pharmacotherapeutic Compass [32], indicating the mean medication usage per adult a day, including the government-imposed discount for patients paid by the pharmacy. Productivity costs will be calculated by means of the friction cost method based on a mean added value of the Dutch working population. The friction costs method takes into account production losses confined to the period needed when it is necessary to replace a sick employee (currently 160 days [31]). In case of uncertainty we will use a conservative estimate (i.e. the lowest cost price). Cost prices will be expressed in Euros on the basis of cost prices of 2011. If necessary, existing cost prices will be updated to 2011 using the consumer price index (CPI) [32]. In this case, discounting is irrelevant as the follow-up period is less than a year.

### Statistical analysis

The power analyses in both trial-based economic evaluations will be based on the RCTs. The primary (base-case) analyses will be performed according to the intention-to-treat principle. This means that data from all participants will be used, regardless of whether they received the intervention or not. For the analyses we will use SPSS statistical software and Excel.

Respondents for whom at least 75% of the data per measurement instrument are available will be included in the analysis. Missing data on item level will be handled using SPSS missing value analysis. Completely missing measurements will be handled using multiple imputation (MI). A baseline analysis will be performed to examine the comparability of groups at baseline for both costs and outcomes. If necessary, methods will be applied to control for differences in baseline [33]. Despite the usual skewed distribution of costs, the arithmetic means is generally considered the most appropriate measure for describing cost data [33]. Therefore arithmetic means (and standard deviations) will be presented. In case of skewed cost data, non-parametric bootstrapping will be used to test for statistical differences in costs between the intervention and control group. Non-parametric bootstrapping is a method based on random sampling with replacement based on the participants' individual data [34]. The bootstrap replications will be used to calculate 95% confidence intervals around the costs (95% CI), based on the 2.5 and 97.5 percentiles. If cost data are distributed normally, t-tests will be used.

ICERs will be calculated for both the CEA and CUA. The ICER will be calculated as follows: ICER = (Ci - Cc)/(Ei - Ec), where Ci is the annual total cost of the new intervention, Cc is the annual total cost of the comparator, Ei is the effects at the 6 month follow-up for the new intervention and Ec is the effect at 6 month follow-up for the comparator.

The robustness of the ICER will be checked by non-parametric bootstrapping. Bootstrap simulations will also be conducted in order to quantify the uncertainty around the ICER, yielding information about the joint distribution of cost and effect differences. The bootstrapped cost-effectiveness ratios will be plotted subsequently in a cost-effectiveness plane, in which the vertical line reflects the difference in costs and the horizontal line reflects the difference in effectiveness. The choice of treatment depends on the maximum amount of money that society is prepared to pay for a gain in effectiveness, which is called the ceiling ratio. Therefore, the bootstrapped ICERs will also be depicted in a cost-effectiveness acceptability curve, showing the probability that the intervention is cost-effective using a range of ceiling ratios.

In addition, to demonstrate the robustness of our base-case findings, a multi-way sensitivity analysis will be performed. In the sensitivity analysis uncertain factors of assumptions in the base-case analysis will be calculated in order to assess whether the assumptions have influenced the ICER, for example by varying cost-prices and volumes between minimum and maximum [34].

#### Cost-of-illness (COI) study as part of the Cohort study

A COI study will be performed to gain insight into care received after stroke and the economic consequences of psychosocial care in particular. A COI study aims at identifying and measuring all the costs of a particular disease, including the direct, indirect and intangible dimensions [35]. This COI study will be embedded in the Cohort study, focussing on the course of quality of life in stroke patients and their informal caregivers, and determining factors predicting quality of life, including pre-stroke health situation factors, stroke-related factors, personal factors and partner factors after stroke. As it is embedded in the Cohort study, the COI study will be related to outcomes of the cohort in a cost-outcome description.

### Sample size calculation

With an inclusion of 500 patients and an expected drop-out rate of 40%, 300 patients should be available for long term analysis (to identify early predictors of long term consequences). For instance, to analyse the course of reintegration and quality of life (linear regression analysis), a total of 300 patients allows regression models with 15 predictors and 15 to 20 subjects per predictor.

### Setting and participants

For the COI study 500 patients will be recruited from the stroke units in 6 participating hospitals in the Netherlands. If present, informal caregivers will also be recruited for the Cohort study to estimate levels of burden, though their presence is not a necessity for the COI study. Written informed consent for participation is obtained after recruitment.

### Timeline

**Figure 4:**
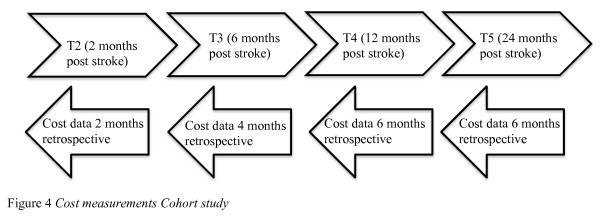
**Cost measurement Cohort study**.

Cost measurements are conducted at 2 months (T2), 6 months (T3), 1 year (T4) and 2 years (T5) post stroke, which is parallel to the measurement points of other questionnaires included in the Cohort study. Retrospective data covering a fixed period prior to measurement points will be extracted from the questionnaires: 2 months at T2, 4 months at T3, 6 months at T4 and 6 months at T5. To avoid recall bias after the two year follow-up, a fixed period of maximum 6 months prior to T5 is chosen. An overview of the timeline for the cost-of-illness cost measurements is presented in Figure 4.

### Outcome measure

For the purpose of the COI study, necessary cost information will be retrieved through a specially designed short cost questionnaire of 11 items covering the costs of psychosocial care after stroke. A simple questionnaire is preferred, for the purpose of the COI study; accordingly the questionnaire has been adapted from previous trial-based economic evaluations studies.

The questionnaire consists of 3 subscales: health care costs, productivity costs and costs of informal care. The dimension of health care costs is captured with 8 items, productivity costs with 2 items and costs of informal care with 1 item. Care received will be measured in absolute numbers, representing visiting times and hours, day and nights spent on health care services and informal care. Due to the fact that the questionnaire is adjusted to be disease-specific every time it is being used, no research has been done on the validity of this questionnaire for stroke research. Nevertheless, the validity of self-reported measurements has been proven earlier in a large multi-centre clinical trial [36].

### Cost analysis

Essentially, there a two approaches to establish the costs of illness: the top-down approach and the bottom-up approach [37]. Scientific publications so far show that the majority of COI studies have been performed using a top-down method, based on calculating the costs of a disease through national databases [8]. The COI study described in this article is designed to use a bottom-up method in which a group of patients who have suffered from a stroke (the cohort population) will be asked what the costs of their disease are, through a specially designed cost questionnaire, instead of using databases. The estimation of costs will be divided into two steps. The first step is estimating the quantity of health inputs used and the second step is to estimate the unit costs of the inputs used [38]. The costs are then estimated by multiplying unit costs by the quantities. One of the main advantages of the bottom-up approach is that detailed data can be obtained regarding costs outside the health care sector such as the productivity losses, which will be investigated in this study [37]. Other advantages are that the bottom-up approach is much more accurate in measuring costs than the top-down approach, which is more likely to present misallocation of costs [38]. For example, national health care expenditures may either under or overestimate the total direct costs, where the bottom-up approach gives a more accurate estimate. Furthermore, a serious problem with the top-down approach is that all costs are attributed to the primary diagnosis [37]. This is a serious problem considering that a relevant part of all hospital discharges involve patients with multiple diagnoses. This is not an issue, and therefore an advantage, with the bottom-up approach. In addition, a follow-up period of 2 years will be used in this study, which will make it possible to distinguish patterns in health care consumption over time.

### Valuation

Within the COI study, the valuation of health care consumption, productivity losses and the costs of informal health care will be similar to the trial-based economic evaluation. This means that the valuation in general will be based upon the Dutch manual for cost analysis in health care research [39].

### Statistical analysis

Missing data and skewed data will be handled similar as in the economic evaluation study, hence respondents for whom at least 75% of the data per measurement instrument are available will be included in the analysis and missing data on item level will be handled using SPSS missing value analysis. Completely missing measurements will be handled using multiple imputation (MI). Arithmetic means will be used to describe cost data, and presented. In case of skewed cost data, non-parametric bootstrapping will be used to test for statistical differences in costs between the intervention and control group. The bootstrap replications will be used to calculate 95% confidence intervals around the costs (95% CI), based on the 2.5 and 97.5 percentiles. If cost data are distributed normally, t-tests will be used.

Concerning the cost measurements, a month of missing data exists between T2 and T3 and 6 months of missing data exists between T4 and T5. To deal with the issue we will use interpolation to construct new data points for these periods. This will be done by calculating mean cost data for the period of 6 months (T3) to 12 months (T4) post stroke and the period of 18 months to 24 (T5) months post stroke. These data will be used to estimate the period between 12 months to 18 months post stroke.

We will do a comparative analysis to estimate differences in costs between 1 year post stroke and 2 years post stroke. Additionally, we will compare cost outcomes and effect outcomes on the primary outcome scales. Furthermore, we will try to determine *high-cost users *and *low-cost users *of health care after stroke. In the latter analysis, *high-cost users *and *low-cost users *will be identified by specific patients' characteristics, costs being the dependent variable and patient characteristics being independent variables.

#### Record linkage study (RLS)

A RLS will be conducted in which the stroke cases registered in the Maastricht University Medical Centre (MUMC) Stroke Register, MUMC Medical Administration, the Mental Health Case Register (MHCR) and GP registrations in the same catchment area, will be linked. Since the available data linking these registers is limited, linking these databases will provide updated and extended information about the current estimates of care consumption and the hypothesis that problems occurring after stroke, such as depression, anxiety and dementia are underestimated and under-diagnosed. The aim of this study is to examine how patterns of health care consumption after stroke (in terms of health care costs, productivity costs, costs of informal care) change during the 5 years after stroke, in comparison with the 5 years before stroke and what the economic impact is of these changes from a societal perspective.

### Sample size calculation

Sample size calculation was not performed because all available patients will be used for data analysis.

### Setting and participants

As mentioned before, four stroke registers will be linked. The MUMC has been chosen because it is evident that the majority of stroke survivors in the area will be admitted to the hospital. Since the MUMC is the only (academic) hospital in that area, it is most likely that stroke patients will be registered in the MUMC database. The MUMC uses a general Medical Administration Database and a specific Stroke Database to collect data on stroke patients. In theory, these databases should overlap 100%, but further research should prove whether this is true. Therefore, we will use both databases capture all stroke cases registered. The organisations involved informed patients about data collection, which means that separate informed consent is not necessary for our record linkage. Approval for the use of these databases for this specific purpose will be requested from the Medical Ethics Committee of the University Hospital Maastricht and Maastricht University.

The MHCR has collected data cumulatively on the psychiatric hospital, the community mental health centre, the psychiatric department of the MUMC, the community psychiatric outreach team, psycho-geriatric nursing homes, sheltered housing, child psychiatric services, services for the mentally impaired, alcohol and drug abuse services etc. The MHCR has also collected demographic and diagnostic data in a region with a population of around 200,000. The region where this research will take place is a city of Maastricht (120,000 inhabitants), a relatively small city in the far south of the Netherlands, and its surrounding area (80,000 inhabitants).

The MUMC is the only hospital in the city of Maastricht and the surrounding area with both a regional and a top-referral care function. In the MUMC, all patients are registered with an ICD-9 or ICD-10 classification, depending on the year of admission.

### Timeline

In this RLS, changes in health care consumption during the five years after stroke in comparison with the five years before stroke and what the economic impact of these changes is, will be studied form a societal perspective. Stroke patients discharged from the MUMC between 2000 and 2005 will be included in this study, which will allow us to analyse patient data from 1995 until 2010 (analyses from5 years before first stroke case until 5 years after last stroke case).

### Cost analysis and valuation

Through anonymous record linkage health care consumption within the 10-year reference period (from 5 years before and after stroke) will be analysed. The first step in the analysis is to examine this consumption before, during and after stroke. In an additional analysis the health care costs will be calculated based on quantities derived from the MHCR and GP registrations. The updated Dutch manual for cost analysis in health care research [39] will be used for the valuation of these health care costs.

### Statistical analysis

The statistical processing program SPSS will be used to link all databases. Databases will be linked anonymously, meaning that specific codes based on different variables will be computed and used to compare patients, instead of specific patient information. To estimate health care consumption before and after stroke, we will divide all stroke patients in 2 equal groups. Therefore, high-cost users of health care after stroke will be compared to low-cost users of health care after stroke. Similar as in the COI-study, we will estimate the differences between high-cost users and low-cost users using regression analysis.

## Discussion

The Restore4Stroke programme aims not only to provide effective interventions, but will also help to identify stroke patients and their informal caregivers at risk of developing long-term problems and refer them to the proper health care professional. The major strength of this programme is that it allows us to combine several powerful research methods into a comprehensive study and to oversee our research outcomes from an aggregate level.

The Restore4Stroke programme is innovative for several reasons. First, the family-centred perspective results in investigating the quality of life in both patients and their informal caregivers. Second, the focus of Restore4Stroke on personal factors as possible determinants of long-term re-integration and on quality of life in a prospective longitudinal design is innovative in comparison with studies focussing on physical and functional consequences [40]. Third, the comprehensive and multidisciplinary character of Restore4Stroke should provide new and necessary information which can serve several different medical sciences and decision makers.

Within Restore4Stroke, the present study, €-Restore4Stroke, will provide new economic insights and evidence for the rehabilitation of psychosocial consequences occurring in the chronic phase after stroke. One of the strengths of the €-Restore4Stroke study is that we include the estimation of health effects on informal caregivers. By adding the CarerQOL questionnaire to our research we not only perform an actual full economic evaluation from a societal perspective, but we are also able to expose the true scope of effects shown by the Self-Management study. Another strength of this study is the inclusion of the RLS, which will give new insights in health care consumption five years before and five years after stroke. To our current knowledge, no RLS has been done recently, although more information has become available about the consequences of stroke. In addition, we will be able to compare our results to a RLS conducted in 2001 [41] and determine what has changed since. Furthermore, we expect that the information we gain from the economic evaluation of the two suggested interventions will add impact on the implementation of these interventions in health care.

## Abbreviations

CBT: Cognitive behavioural therapy; CEA: Cost effectiveness analysis; CI: Confidence interval; COI: Cost of illness; CPI: Consumer price index; CUA: Cost utility analysis; DDD: Daily defined dosage; EQ-5D: EuroQol 5 dimensional; GP: General practitioner; HADS: Hospital anxiety depression scale; ICER: Incremental cost effectiveness ratio; MHCR: Mental health case register; MUMC: Maastricht University Medical Centre; QALY: Quality adjusted life year; RCT: Randomized controlled trial; RLS: Record linkage study; RNH: General practitioners registry (RegistratieNet Huistartspraktijken); SMI: Self-management intervention; UK: United Kingdom; UPCC: Utrecht proactive coping scale; VAS: Visual analogue scale.

## Competing interests

The authors declare that they have no competing interests.

## Authors' contributions

MvE is the primary investigator of the €-Restore4Stroke study. MvE is responsible for the data collection for €-Restore4Stroke; he will monitor and execute the study. CvH and SE supervise the €-Restore4Stroke study. All authors read and approved the final manuscript.

## Current study status

The Restore4Stroke research programme started in September 2010. The €-Restore4Stroke study has almost finished its preparation phase and will start its data collection parallel to the RCTs and cohort data collection to which it is attached. The Cohort study, the Augmented CTB study and the Self-Management study received approval from the Medical Ethics Committee.

## Pre-publication history

The pre-publication history for this paper can be accessed here:

http://www.biomedcentral.com/1471-2458/12/122/prepub
